# The evolution of the duckweed ionome mirrors losses in structural complexity

**DOI:** 10.1093/aob/mcae012

**Published:** 2024-02-02

**Authors:** Kellie E Smith, Min Zhou, Paulina Flis, Dylan H Jones, Anthony Bishopp, Levi Yant

**Affiliations:** School of Life Sciences, University of Nottingham, Nottingham NG7 2RD, UK; School of Biosciences, University of Nottingham, Sutton Bonington LE12 5RD, UK; School of Biosciences, University of Nottingham, Sutton Bonington LE12 5RD, UK; School of Biosciences, University of Nottingham, Sutton Bonington LE12 5RD, UK; School of Biosciences, University of Nottingham, Sutton Bonington LE12 5RD, UK; School of Life Sciences, University of Nottingham, Nottingham NG7 2RD, UK; Department of Botany, Faculty of Science, Charles University, Prague, Czech Republic

**Keywords:** Vestigiality, duckweed, ionomics, evolution, ICP-MS, *Spirodela*, *Landoltia*, *Lemna*, *Wolffiella*, *Wolffia*

## Abstract

**Background and Aims:**

The duckweeds (Lemnaceae) consist of 36 species exhibiting impressive phenotypic variation, including the progressive evolutionary loss of a fundamental plant organ, the root. Loss of roots and reduction of vascular tissues in recently derived taxa occur in concert with genome expansions of ≤14-fold. Given the paired loss of roots and reduction in structural complexity in derived taxa, we focus on the evolution of the ionome (whole-plant elemental contents) in the context of these fundamental changes in body plan. We expect that progressive vestigiality and eventual loss of roots might have both adaptive and maladaptive consequences that are hitherto unknown.

**Methods:**

We quantified the ionomes of 34 accessions in 21 species across all duckweed genera, spanning 70 Myr in this rapidly cycling plant (doubling times are as rapid as ~24 h). We related both micro- and macroevolutionary ionome contrasts to body plan remodelling and showed nimble microevolutionary shifts in elemental accumulation and exclusion in novel accessions.

**Key Results:**

We observed a robust directional trend in calcium and magnesium levels, decreasing from the ancestral representative *Spirodela* genus towards the derived rootless *Wolffia*, with the latter also accumulating cadmium. We also identified abundant within-species variation and hyperaccumulators of specific elements, with this extensive variation at the fine (as opposed to broad) scale.

**Conclusions:**

These data underscore the impact of root loss and reveal the very fine scale of microevolutionary variation in hyperaccumulation and exclusion of a wide range of elements. Broadly, they might point to trade-offs not well recognized in ionomes.

## INTRODUCTION

The duckweeds (Lemnaceae) consist of 36 species exhibiting broad variation, including, in recently derived species, the progressive evolutionary loss of a fundamental plant organ, the root. This progressive loss of roots is accompanied by an overall reduction in vascular tissues in derived taxa. Given the paired loss of roots and reduction in structural complexity, we focus here on the evolution of the ionome and place it in the context of these fundamental changes in body plan.

**Fig. 1.**
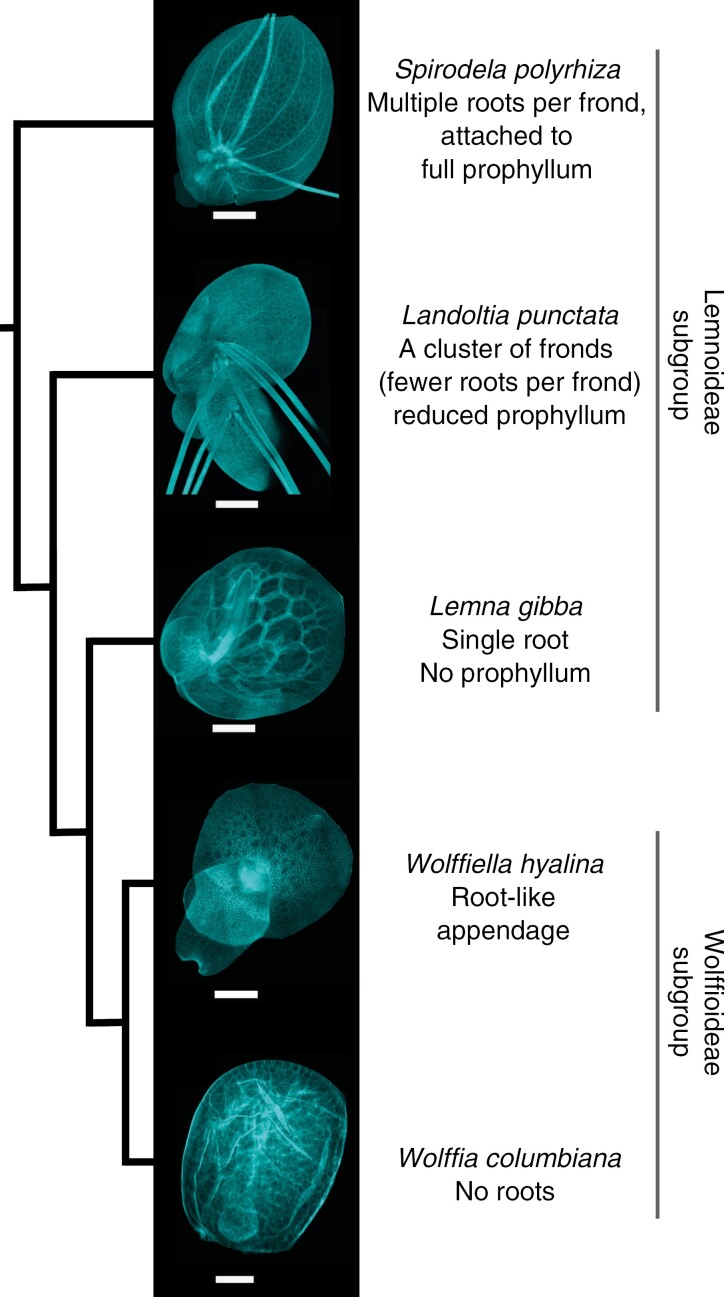
Trajectory from ancestral root-harbouring duckweeds, via vestigiality, to root loss. Ancestral form (above) represented by Lemnoideae: *Spirodela*, *Landoltia* and *Lemna*. Derived from (below) shown in Wolffioideae subgroup genera *Wolffiella* and *Wolffia*. All samples were cleared, stained with Fluorescent Brightener 28 (calcofluor) following the protocol described by Kurihara *et al.* (2015) and imaged on a Leica TCS SP5 confocal microscope. Scale bars: *Spirodela* and *Landoltia*, 1000 µm; *Lemna* and *Wolffiella*, 500 µm; *Wolffia*, 100 µm. Cladogram schematic topology is based on Tippery *et al.* (2015).

Consisting of five genera progressively differing in the number of roots and vascular complexity, the duckweeds present broad variation in highly simplified body plans (Fig. 1). The earliest diverged lineages, *Spirodela* and *Landoltia* (Fig. 1, top), were originally both considered *Spirodela*, but are now recognized as distinct (Les and Crawford, 1999; Les *et al.*, 2002; Bog *et al.*, 2015). The three more recently diverged genera, *Lemna*, *Wolffiella* and *Wolffia*, represent novel forms, with progressively diminished roots and reduced vascular tissues (called nerves) or none at all (Fig. 1, bottom; Appenroth *et al.*, 2013; Tippery *et al.*, 2015). The divergence time between rooted *Spirodela polyrhiza* and rootless *Wolffia australiana* is estimated at 70 Myr (Park *et al.*, 2021). Since this divergence, ≥36 duckweed species have formed (Appenroth and Sree, 2020; Bog *et al.*, 2020), which vary 14-fold in genome sizes (Hoang *et al.*, 2019). The smallest is an *Arabidopsis*-scale 158 Mb genome in *Spirodela polyrhiza* (Wang *et al.*, 2011; An *et al.*, 2018), with the largest genomes in the derived *Wolffia*, which exhibit a radically simplified body plan, diminished vasculature and no roots (Fig. 1 bottom row; Park *et al.*, 2021; Yang *et al.*, 2021).

In contrast to vascular land plants, duckweeds have miniscule bodies in direct contact with water and limited to non-existent root systems. This results in small distances for ion translocation (Zhang *et al.*, 2009). However, the relative differences in translocation distance can be large: frond sizes of *Spirodela* are >1 cm, but in *Wolffia* only <1 mm. Duckweed roots are considered adventitious, lacking lateral roots and root hairs (An *et al.*, 2019). Root-forming species have flexibility in their root systems, which can develop or elongate in stressful situations or drop off (Landolt, 1986). Root functions in anchorage, aggregation to form duckweed mats and aiding dispersal by attachment have all been proposed (Cross, 2017; Ware *et al.*, 2023). In the highly derived Wolffioideae, the shrinking of body size and complete root loss have evolved to maximize growth rate, improve mobility and enhance adaptability to changing environments (Wang *et al.*, 2010; Michael *et al.*, 2020; Yang *et al.*, 2021). We expect that duckweeds, representing this unique example of progressive root reduction through to complete loss, will illustrate a gradient of phenotypic changes resulting in altered internal macronutrient and trace element compositions (Ware *et al.*, 2023).

At the fine scale, duckweed habitats differ in their availability of elements; thus, adaptation of accessions to their environments can occur through different elemental storage and exclusion strategies (Mkandawire and Dudel, 2007; Zhang *et al.*, 2009; Van Dam *et al.*, 2010; Lahive *et al.*, 2011). The tolerance of duckweed to elemental extremes is an important trait driving adaptive (and sometimes strongly invasive) strategies in the wild (Wang, 1991; Naumann *et al.*, 2007; Ekperusi *et al.*, 2019). To date, however, the tolerance of only a handful of duckweed accessions to external elemental concentrations has been assessed, with reports focusing on growth vigour vis-à-vis single elements in *Lemna* and *Landoltia* species. Studies quantifying elemental composition are rare, with the broadest study looking at only a single genus, *Wolffia*, with 11 species being assessed (Appenroth *et al.*, 2018). We collected existing reports of duckweed elemental variation; however, serious confounding factors plague interpretation of different studies, owing to discordant methods and quantification (Table 1).

**Table 1.** Elemental tissue concentration of duckweeds gathered from the literature. Elements are ordered by type (macro, micro, trace elements and heavy metals) reported from the literature and included in our experiment.

Element	Species	Fold variation (literature)	Fold variation (this study, 21 species)
P	*Wolffia* spp.	1.7^1,2^	2.4
K	*Lemna* spp., *Wolffia* spp.	2.4^1,2^	3.3
Ca	*Lemna* spp., *Wolffia* spp.	3.3^1,2^	11.4
Mg	*Lemna* spp., *Wolffia* spp.	3.1^1,2^	19.5
Na	*Lemna* spp., *Wolffia* spp.	29.5^1,2^	27.4
Fe	*Lemna* spp., *Wolffia* spp.	21.8^1,2^	111.0
Zn	*Lemna gibba*, *Lemna minor*, *Landoltia punctata*, *Wolffia* spp.	87.3^1,3,4,5^	149.6
Mn	*Spirodela polyrhiza*, *Wolffia* spp.	27.3^1,6^	4.5
Cu	*Lemna trisulca*, *Lemna gibba*, *Lemna minor*, *Wolffia* spp.	15.7^1,7,8,9^	7.6
Cd	*Landoltia punctata* 6001, *Lemna minor*, *Lemna gibba*, *Spirodela polyrhiza* sp., *Wolffia globosa*	5900^1,10,11,12,13,14^	27.3

^1^ Appenroth *et al.* (2018). ^2^Mkandawire and Dudel (2007). ^3^Van Steveninck *et al.* (1992). ^4^Khellaf and Zerdaoui (2009). ^5^Lahive *et al.* (2011). ^6^Liu *et al.* (2017). ^7^Prasad *et al.* (2001). ^8^Leblebici *et al.* (2010). ^9^Landolt and Kandeler (1987).

**Fig. 2.**
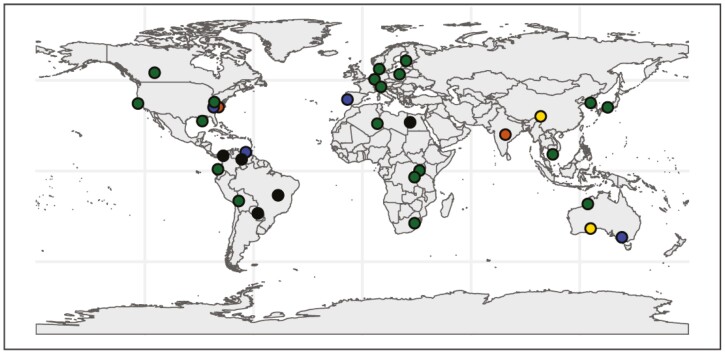
Sampling of worldwide duckweeds for ionomic panel. Dots indicate sample origin locations: *Lemna* = green, *Landoltia* = yellow, *Spirodela* = black, *Wolffiella* = orange and *Wolffia* = blue. Duckweeds were derived from the Landolt collection, now housed in Milan.

Here, we bridge this gap, reporting whole-plant ionome compositions in 34 duckweed accessions spanning 21 species and representing the worldwide range of all five duckweed genera (Fig. 2; Supplementary Data Table S1). We place these data into an evolutionary context, focusing on 11 key macro-, micro- and trace elements, contrasting microevolutionary variation (accession-level, within-species variation) with macroevolutionary trends (between genera). These results reveal extensive ionomic variation at both the within-species and between-genus levels, with particularly clear trends for differences in Ca and Mg accumulation, in addition to possible excess Cd accumulation in the rootless *Wolffia/Wolffiella*. We discern a broad evolutionary trajectory towards very low levels of essential Ca and Mg, in addition to increased Cd accumulation, in the recently derived rootless species. This suggests a potentially deleterious consequence associated with the root loss and body-wide reduction in vasculature.

## MATERIALS AND METHODS

### Plant growth and care

Duckweed accessions were grown in axenic conditions from single isolates or from five to ten individuals, depending on the size of duckweeds, in 100 mL of nutrient medium (N medium) in individual sealed sterile glass conical flasks. Duckweeds were sourced from the Landolt Collection (now housed in Milan). The N medium was described by Appenroth *et al*. (1996) [KH_2_PO_4_, 0.15 mm; Ca(NO_3_)_2_, 1 mm; KNO_3_, 8 mm; MgSO_4_, 1 mm; H_3_BO_3_, 5 µm; MnCl_2_, 13 µm; Na_2_MoO_4_, 0.4 µm; and FeEDTA, 25 µm]. Concentrations of elements in the supplied N medium, including the presence of other trace elements, were measured by inductively coupled plasma mass spectrometry (ICP-MS) and are presented in the Supplementary Data (Dataset S1). Weekly media changes were performed, with rinses in Milli-Q (Millipore) water to regulate nutrient composition availability. Plants were grown at 100 µmol m^−2^ s^−1^ under broad-spectrum (white) LED lights at 22 °C/18 °C with a 16 h day/night cycle. Four-week-old duckweed cultures were washed on plastic sieves using a three-step protocol for 2 min each of Milli-Q (Millipore) water, CaCl_2_ and Na-EDTA and harvested into individual samples from flasks of individual populations. These were harvested for ICP-MS analysis on day 1, 3 and 5 after media change, *n* = 6 per time point. Four-week-old cultures are clonally reproduced and therefore suitable replicates, given the very low generational variation and low mutation rates shown in duckweed mutation accumulation experiments (Xu *et al.*, 2019).

### Imaging and microscopy

All samples were cleared, then stained with Fluorescent Brightener 28 (calcofluor) following the protocol described by Kurihara *et al.* (2015) and imaged on a Leica TCS SP5 confocal microscope. In short, plants were cleared, based on the ClearSee procedure described by Kurihara *et al.* (2015), with slight modification. Given that fluorescent markers were not being used, plants were fixed overnight in ethanol and acetic acid (3:1 v/v) rather than paraformaldehyde, because this reduced the toxicity and requirement for vacuum infiltration, which can be damaging to the air spaces. Plants were then rinsed three times with reverse osmosis water and left for 30 min, after which the reverse osmosis water was replaced with ClearSee solution (10 % xylitol, 15 % sodium deoxycholate and 25 % urea; Kurihara *et al.*, 2015) and left to clear for 2 weeks. Before imaging, plants were stained for 1 h with calcofluor in ClearSee (100 μg mL^−1^), then washed in ClearSee for 1 h. Imaging was carried out using a confocal laser scanning microscope (Leica SP5), using a 405 nm diode laser at 12 % and hybrid detector with a range of 440–450 nm, gain of 25 % and pinhole of 0.5 Airy units.

### Quantification of elemental tissue concentrations

For ICP-MS, we used a method adapted from the study by Danku *et al*. (2013). Briefly, 5–20 mg (fresh weight) was harvested per sample, placed in Pyrex test tubes and dried at 88 °C for 24 h. The dry weight was recorded, then 1 mL concentrated trace metal grade nitric acid Primar Plus (Fisher Chemicals) spiked with an internal standard was added to the samples, which were digested further in DigiPREP MS dry block heaters (SCP Science; QMX Laboratories) for 4 h at 115 °C. Before the digestion, 20 µg L^−1^ of indium (In) was added to the nitric acid as an internal standard for assessing errors in dilution, variations in sample introduction and plasma stability in the ICP-MS instrument. Then 0.5 mL of hydrogen peroxide (Primar, for trace metal analysis, Fisher Chemicals) was added to the samples and they were digested for additional 1.5 h at 115 °C. After digestion, samples and blanks were diluted to 10 mL with Milli-Q (Millipore). Direct water and elemental analysis was performed using an ICP-MS, PerkinElmer NexION 2000, with 22 elements monitored (Li, B, Na, Mg, P, S, K, Ca, Cr, Mn, Fe, Co, Ni, Cu, Zn, As, Se, Rb, Sr, Mo, Cd and Pb) in the collision mode (He). To correct for variation between and within ICP-MS analysis runs, liquid reference material was prepared using pooled digested samples and run after every nine samples in all ICP-MS sample sets. The calibration standards were prepared from single element standard solutions (Inorganic Ventures; Essex Scientific Laboratory Supplies Ltd, Essex, UK). Sample concentrations were calculated using an external calibration method within the instrument software. Further data processing, including calculation of final elements concentrations (in milligrams per kilogram), was performed in Microsoft Excel. Log_10_-transformations, *z*-score calculations and graphical representation were performed using R (v.3.0.2 ‘Frisbee Sailing’; R Development Core Team, 2023; see http://www.R-project.org), and RStudio v.1.0.136 (RStudio Team, 2020) was used for all statistical analyses. To calculate relationships between elements, the corrplot package (McKenna *et al.*, 2016) was used in R with Pearson correlations on log_10_-transformed data.

## RESULTS

### Broad scale evolution of the ionome

**Fig. 3.**
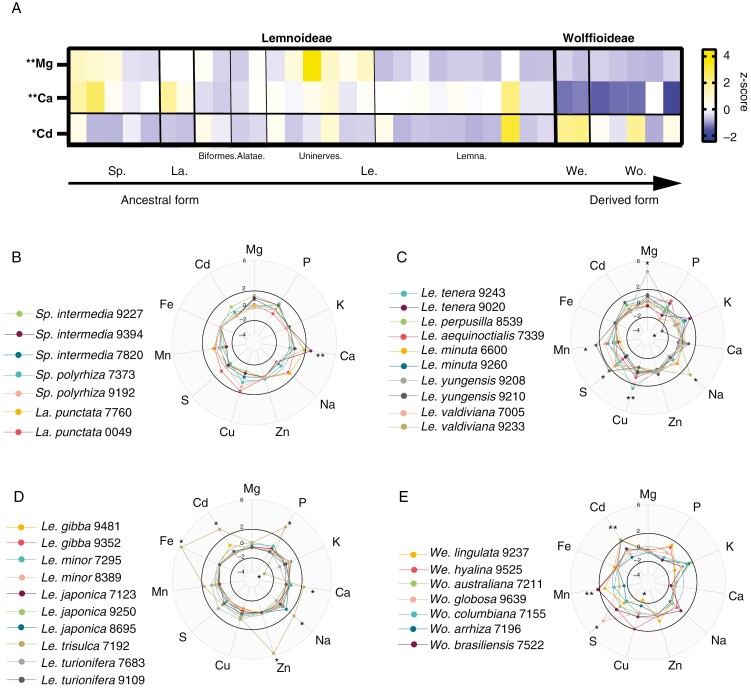
The evolution of the duckweed ionome across genera, species and accessions. (A) Relative levels of elemental accumulation across rootless and rooted subgroups, respectively. The heat map is coloured by *z*-scores for the four most differentially accumulated elements. Significant differences were determind by ANOVA with Tukey’s post-hoc test set at **P < 0.01 and *P < 0.05 between Wolffioideae and Lemnoideae. The *z*-scores (number of standard deviations away from the mean) were generated for each element using log_10_-transformation of values (in milligrams per kilogram) on day 5. The *x*-axis is arranged with basal forms on the left and derived forms on the right. Separating lines indicate genus and subgroup boundaries. We. = *Wolffiella* (2), Wo. = *Wolffia* (5), Le. = *Lemna* (20), La. = *Landoltia* (2) and Sp. = *Spirodela* (5). Within *Lemna*, sections *Biformes*, *Alatae*, *Uninerves* and *Lemna* are marked from left to right. (B–E) Radar plots showing differences in ionome profiles between individual accessions: (B) *Spirodela* and *Landoltia*; (C) *Lemna* sections *Biformes*, *Alatae* and Uninerves; (D) *Lemna* section *Lemna*; and (E) *Wolffiella* and *Wolffia* species. Species are ordered in the panels according to Tippery *et al.* (2015), from the most ancestral representative at the top left to the most derived at the bottom right. Numbers after species represent clone numbers. Asterisks represent a significant increase or decrease of ±2 relative to all normalized element concentrations for all species based on the mean and SD. The complete dataset of 17 elements and three time points is given in the Supplementary Data (Dataset S2).

We focus on ionomes from day 5 after media change (Fig. 3), which is representative of other time points (none of the 11 elements upon which we focus was significantly different across days by ANOVA). The full raw dataset is given in the Supplementary Data (Dataset S2); elements we considered for further analysis are shown in the Supplementary Data (Fig. S1). Concentrations were consistent for all elements for all accessions between time points except for a handful of elements in certain accessions depicted in the Supplementary Data (Fig. S2). These exceptions show a small minority of accessions decreasing in K, Ca, Fe and Cd and others still increasing (e.g. Ca, Cu and Fe). For accumulators showing the latter pattern, such as *Spirodela intermedia* 9227, the maximum concentration capacity of Ca on day 1 after media changes was still not reached, despite high nutrient provision throughout a 4-week experimental period, and the accession could still prolong uptake.

**Fig. 4.**
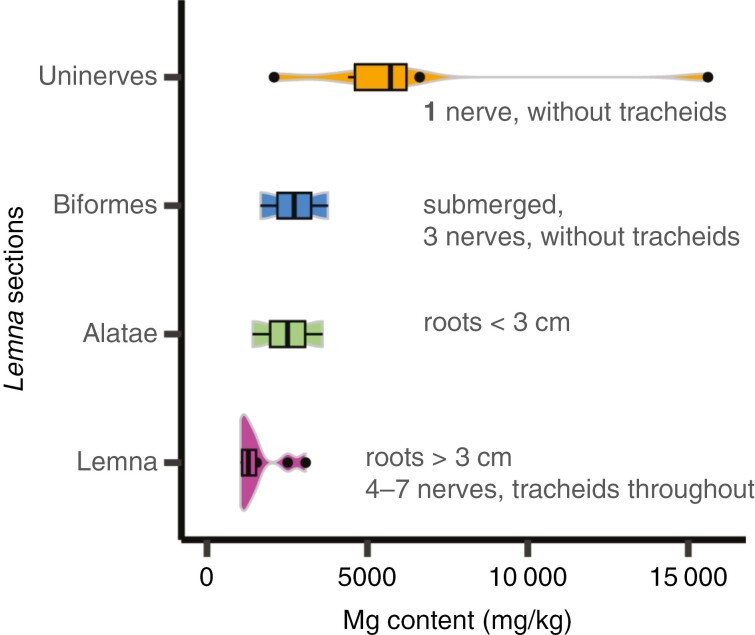
Increased Mg content mirrors the reduction of frond vasculature within *Lemna*. The four sections of *Lemna* represent the highest Mg content in the species with most reduced vasculature for section *Uninerves*, with transitional sections *Biformes* and *Alatae* and the most developed frond vasculature in section *Lemna*, with reduced Mg. The Mg content is plotted from day 5 averaged values for each accession within each section: *Uninerves*, *n* = 6; *Biformes*, *n* = 2; *Alatae*, *n* = 2; and *Lemna*, *n* = 10. Sections are ordered and described according to Landolt (1986) and Tippery *et al.* (2015). Violin plots represent the spread of data for each group, with the middle line plotting the mean.

In the overall dataset of 34 accessions, the broadest contrast observed was between the Lemnoideae and Wolffioideae (rooted and rootless, respectively) for Ca, Mg and Cd accumulation (Fig. 3A). All ancestral representatives of (rooted) Lemnoideae (*Spirodela*, *Landoltia* and *Lemna*) consistently exhibited two to three times higher Ca content relative to the derived rootless Wolffioideae (*P* ≤ 0.01; log_10_, ANOVA with Tukey’s post-hoc test). Likewise, on average, Mg accumulation was 1.8 times higher in the rooted species relative to the rootless *Wolffia* and *Wolffiella*. Ca and Mg showed a positive correlation (Table 2; Supplementary Data Figs S3 and S4). We observed further variation for Mg in the *Lemna* genus, where there emerged a gradient of Mg accumulation across *Lemna* sections (Figs 1 and 3A, D). The highest Mg levels were in the *Uninerves* section (Figs 3A and 4), which includes the invasive *Lemna minuta* and *Lemna yungensis* (now *Lemna valdiviana*), as described by Tippery *et al.* (2015) and Bog *et al.* (2020), both alien within Europe (Kirjakov and Velichkova, 2016; Ceschin *et al.*, 2018). This association of Mg accumulation with increased root vasculature (and with reduced frond vasculature in *Lemna*) stood in strong contrast to the uniformly very low Mg in rootless Wolffioideae. Cadmium concentrations varied significantly between rooted and non-rooted duckweeds (Fig. 3A; *P* < 0.05; log_10_, ANOVA with Tukey’s post-hoc test) in a manner inverse to Ca and Mg. The unrooted Wolffioideae species (especially *Wolffiella*) showed the highest Cd concentrations. Only the submerged *Lemna trisulca* exhibited Cd comparably high to the Wolffioideae (Fig. 3).

**Table 2.** Mg and Ca were correlated strongly and positively with various elements, whereas K was negatively correlated. Element pairs were significantly correlated across 34 duckweeds at three time points. The *R* values correspond to positive or negative Pearson correlations derived from log_10_-transformed data for eight elements. Data are given to two decimal places.

Element	*R*
Fe/K	−0.76
Zn/K	−0.72
P/K	−0.67
Mn/K	−0.59
Mg/Ca	0.59
Fe/Mn	0.58
Zn/Mn	0.58

Rootless species exhibiting variation in at least two elements included *Wolffiella lingulata*, *Wolffiella hyalina* and *Wolffia brasiliensis* (Fig. 3E). In contrast, the species in our panel from the multi-rooted, more ancestral duckweed representatives, *Spirodela* and *Landoltia*, showed the greatest ionomic consistency across all accessions (Fig. 3B). *Spirodela* species had the highest tissue content of Ca in our panel, but other elements were not as variable between accessions.

### *Fine-scale ionome variation and identification of extreme accumulators in* Lemna

We observed the greatest within-genus ionome variation in the *Lemna* genus (*n* = 20 accessions, six biological replicates of each; Fig. 3C, D). *Lemna* also harboured several extreme accumulators, each standing as outliers for the accumulation of three or more elements. *Lemna trisulca* 7192 has a submerged growth pattern and accumulated the greatest number of elements in amount and number from the panel, showing very high tissue concentrations of four essential elements (P, Ca, Zn and Fe), in addition to Cd, and low K levels (Fig. 3D). *Lemna yungensis* 9210 accumulated high S and Mn and also exhibited low K (Fig. 3C). The K levels trended negatively against the enhanced accumulation of other macro- and microelements in both *Le. trisulca* and *Le. yungensis* and across our panel as a whole (Table 2; Supplementary Data Fig. S4).

### *Fine-scale ionome variation between* Lemna *species*

**Fig. 5.**
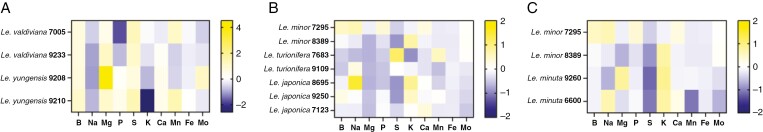
Elements high in N medium show limited differences in internal ionomes between pairs of *Lemna* species. (A) *Lemna yungensis* (now merged with *Lemna valdiviana*) and *Le. valdiviana* accessions. (B) *Lemna minor*, *Lemna turionifera* and their interspecific hybrid species, *Lemna japonica*. (C) Accessions of cosmopolitan *Lemna minor* and invasive European alien *Lemna minuta*. Heat maps for *z*-scores from day 5 are presented for each accession. Ten elements were selected based on those intentionally added and present in the highest concentrations in N medium. The *z*-scores ± 2SD represent a significant increase or decrease relative to all normalized elements.

We noted variation at the level of several accession pairs, most obviously between *Le. yungensis* accessions (Fig. 3C). Notably, *Le. yungensis* 9208 greatly accumulated Mg, and *Le. yungensis* 9210 exhibited extreme accumulation of S and Mn, but low K. When comparing *Le. yungensis* with *Lemna valdiviana* clones, none of the accessions showed large differences in ionomes between ten elements, with consistent levels of B and S (Fig. 5A). Comparing *Lemna minor* with *Lemna turionifera* and their interspecific hybrid *Lemna japonica*, *Le. japonica* accessions had lower Mo and a slight increase in Na and K in specific *Le. japonica* clones (Fig. 5B); however, neither of these ionome changes was significant in comparison to the whole duckweed panel. When contrasting native European *Le. minor* clones with invasive European *Le. minuta*, we saw clone-level variation in some elements, but none varied significantly from the overall population by as much as one SD (Fig. 5C).

## DISCUSSION

The broad variation we observed in duckweed ionomes at levels of genera, species and sister accessions is presumably attributable, in large part, to both morphological differences and adaptation to micro-environments. The most robust differences were at the genus level for Ca, Mg and Cd. The accumulation difference for Ca is perhaps explained, in part, by a storage mechanism as calcium oxalate (CaOx) within frond crystal ultrastructures in rooted genera, in the fronds of *Spirodela* and *Lemna* (Landolt and Kandeler, 1987) and in the root of *Le. minor* (Franceschi, 1989; Mazen *et al.*, 2003). In *Le. turionifera*, Ca influxes through roots and is stored in both fronds and roots, and in exceptional cases it can also be effluxed out of roots (Ren *et al.*, 2022). In contrast, Wolffioideae species have soluble Ca in cell sap and accordingly also cannot store excess Ca in the roots (Landolt and Kandeler, 1987; Appenroth *et al.*, 2017); thus Ca and Mg might be lower in Wolffiodeae because they lack roots as a storage organ. Given that Ca was kept sufficiently available in our experiment through media refreshes, and rooted duckweeds use their roots as an additional storage compartment (Ren *et al.*, 2022), this might result in overall higher accumulation when compared with their rootless counterparts.

Given the broad contrasts in Ca between genera, it is interesting to consider these results alongside the importance of roots for elemental uptake and segregation of individual elements between the frond and root in duckweed species. The excision of roots makes only a modest change to the frond ionome, showing that roots are vestigial and overall not required for nutrient uptake in replete media conditions (Ware *et al.*, 2023). This supports the notion that duckweed roots might be adventitious (Landolt, 1986; An *et al.*, 2019). Although, surprisingly, removal of roots increased elemental composition in some cases (Ware *et al.*, 2023), the picture is more complicated, in that rootless species do not naturally exhibit elevated Mg or Ca in our data, indicating evolutionary adjustment of ion homeostasis upon root loss. The *Wolffia* genome harbours a derived complement of Ca export and cell wall-thickening genes, possibly minimizing potential for apoplastic transport, which, coupled with inability for storage as CaOx, results in less specialized mechanisms to manoeuvre and store Ca content overall (Michael *et al.*, 2020). In contrast, clones of *Le. aequinoctialis*, *Le. minuta* and *Le. minor* exhibit marked Ca accumulation (storage) to alleviate Mg toxicity from a contaminated mine and in high Mg:Ca ratio media or wastewater (Van Dam *et al.*, 2010; Paolacci *et al.*, 2016; Walsh *et al.*, 2020). This suggests specific adaptation of Ca storage and transport mechanisms to particular ionomic challenges.

The Mg gradient across *Lemna* species is not necessarily correlated with strict overall inferred ancestral and derived forms (Wang *et al.*, 2011; Tippery *et al.*, 2015) and root vascular complexity is not sufficiently varied between rooted duckweeds to account for this (Ware *et al.*, 2023). Instead, higher specific Mg uptake in the *Uninerves* section of *Lemna* might be associated with their reduced frond vascular complexity (Figs 3A and 4). With typical frond nerves numbering ≤16 in in *Spirodela* and between three and seven in other *Lemna* species (Les *et al.*, 2002), only one nerve is present in *Le. yungensis* and *Le. minuta*, with *Le. yungensis* (now *Le. valdiviana*) having the longer nerve of the two (Landolt, 1980; Crawford *et al.*, 1996). It is thought that this simplified vascular system might contribute to their invasive status (Kirjakov and Velichkova, 2016; Kadono and Iida, 2022). Reduced vascular complexity and ionomic differences could also offer enhanced potential for adaptation to varied environments, showing higher Mg tolerance (Paolacci *et al.*, 2016) and possibly, therefore, survival in hard water.

Although some variation in mineral content among *Wolffia* species has been reported by Appenroth *et al.* (2018), *Wolffiella* have received little attention and can be under-reported owing to clones having restricted biogeography and not being readily available (Landolt, 1986; Kimball *et al.*, 2003). Therefore, multi-elemental compositions of rooted and rootless duckweeds have not been compared directly before. In this respect, we see relative accumulation of Cd, especially in *Wolffiella* compared with the rooted species. This is somewhat surprising, because it might be expected that Cd accumulation would be detrimental to minuscule plants with no root segregation away from photosynthetically active tissue. We note, however, that *Wolffia* species also exhibit tolerance to As and have been considered as candidates for phytoremediation, accumulating more than Lemnoideae (Zhang *et al.*, 2009). Additionally, there is good evidence that *Wolffia* has moderate tolerance to Cd and increased accumulation capacity even in extreme concentrations (>200 µm). In fact, a handful of *Wolffia* species show Cd uptake in as little as 30 min from solution via apoplastic transport, which increases linearly with Cd concentration (Boonyapookana *et al.*, 2002; Xie *et al.*, 2013). We therefore speculate that loss of roots could have reduced control of heavy metal uptake whilst, at the same time, root loss removes a potential mechanism of uptake and a storage compartment available to rooted species (Verma and Suthar, 2015; Ma *et al.*, 2023; Zheng *et al.*, 2023). Wolffioideae perhaps evolved higher tolerance mechanisms to Cd toxicity, such as compartmentalization to vacuoles and complexation via conjugates (Schreinemakers, 1986). Although Cd was not supplied in a dedicated quantity in N medium preparation, we quantified the presence of Cd by ICP-MS in the media used (Supplementary Data Dataset S1) and suggest that this comes from chemical impurities, as indicated by Appenroth *et al.* (2018). We infer that *Wolffioideae* species might have a potential for heavy metal accumulation at higher dosages than those given here, perhaps also in the wild through adaptation to contaminated habitats (Zhang *et al.*, 2009).

Our results showed that the genus with the greatest diversity of specific accumulators was *Lemna*. The *Lemna* accessions with most extreme ionomes, *Le. trisulca* 7192 and *Le. yungensis* 9208, also harbour the most divergent root architecture, in comparison to other species of *Lemna*. *Lemna trisulca* is characterized by a submerged growth habit but smaller cortical cells, giving a thin, reduced root compared with other *Lemna* species, and *Le. yungensis* 9208 often displays an additional layer of cortical cells and irregularly large extracellular airspaces in the root cortex (Ware *et al.*, 2023). Thus, these differential root vasculature components, coupled with minimal frond vasculature, might play a role in producing the contrasting elemental profiles observed. Both *Le. trisulca* and *Le. yungensis* accumulated >1000 mg kg^−1^ dry weight for several elements and can therefore be considered hyperaccumulators (Zayed *et al.*, 1998; Zhang *et al.*, 2009). For this reason, these two species might have potential to be used in combination to alleviate multi-elemental toxicity in watercourses. *Lemna trisulca* accumulated greater Zn and Cd than floating species, possibly because of increased absorption through submerged fronds. Although *Le. trisulca* had the greatest variation overall and maximal micronutrient levels, the associated high Cd accumulation might be problematic for any applications in nutrition. It is also unclear whether this trait is common in other *Le. trisulca* accessions owing to limited availability of clones in stock centres; however, this species has previously been noted for its Cd accumulation potential (Kara and Kara, 2005).

A greater appreciation for duckweed variation in the micronutrients Ca, Mg, Fe and Zn is clear from our study, with particular accessions acting as hyperaccumulators for multiple nutritionally relevant elements. This is not the case for trace elements, such as Na and Cu (and especially Mn and the heavy metal Cd), for which the variation in tissue concentration was less dramatic than seen in other reports (Table 1). This is probably attributable to the combined effect of low presence of these elements in our supplied media or that comparisons across literature are confounded by variables disallowing truly quantitative comparisons between studies. This is particularly evident for Cd, which we supplied in only trace amounts (Supplementary Data Dataset S1), whereas external Cd concentrations vary 500-fold between studies.

Synthetic biology, including the tailoring of ionomic profiles in duckweeds, is an important goal of the duckweed research community (Lam and Michael, 2022). Interestingly, the *Spirodela* genome sizes are the smallest and the ionomes the least variable among all duckweeds here (Wang *et al.*, 2011; An *et al.*, 2018); additionally, the amenability of *Spirodela* to genetic transformation (Yang *et al.*, 2018*a*, *b*) makes it a strong candidate as a minimal scaffold for synthetic biology. We also suggest that because their ionomic profiles are so variable, the species harbouring larger genomes will be particularly valuable to mine natural variation to inform transgenic approaches in the smaller, highly tractable *Spirodela* genome.

For the fine-scale variation between *Lemna* species of interest, the vast ionome differences between *Le. yungensis* 9208 and 9210 can be ascribed best to local adaptation. Given that these accessions are closely related and were both originally isolated from the same region in Bolivia, one might expect more similar ionome profiles, but instead our data show that duckweeds exhibit strongly contrasting local variation in elemental uptake. Interestingly, this region of Bolivia is reported to be atypically harsh for duckweed, growing on sheer rock faces with waterfall spray with low nutrient availability (Landolt, 1998). It will be valuable to characterize *Le. yungensis* species further, in order to determine the genetic basis for their adaptation to specialized habitats. Given that *Le. yungensis* and *Le. valdiviana* showed no other significant internal differences between ten elements, this supports their unification as one species owing to lack of genetic differentiation (Bog *et al.*, 2020). *Lemna minuta* is an invasive species in introduced regions with ecological significance (Ceschin *et al.*, 2018), as an opportunist species in replete N and P with additional higher Mg tolerance (Njambuya *et al.*, 2011; Paolacci *et al.*, 2016; Ceschin *et al.*, 2020) one would expect drastic differences in the ionome in comparison to *Le. minor*. Despite this, there were no clear pattern differentiating two *Le. minuta* from two *Le. minor* clones grown in nutrient-rich medium (N medium; Appenroth *et al.*, 1996; measured here in Supplementary Data Dataset S1). Elemental differences seem to be at the clonal level, and opportunism therefore probably depends on unique situations in the wild. Recent data classified *Le. japonica* as a hybrid between *Le. minor* and *Le. turionifera* (Braglia *et al.*, 2021; Volkova *et al.*, 2023). Hybrid *Lemna japonica* clones had slightly reduced Mo compared with their parents, and one clone had significantly higher Na. It could be that hybridization might result in ionome differences important for altered adaptation to varied environments, as found in other plant species (Arnold *et al.*, 2016; Wong *et al.*, 2022). Taken together, between these groups of *Lemna* species, subtle interspecies differences for elements were clear. The physiological differences between species and their clones in light of genetic differences deserve future attention in duckweed.

### Conclusions

Here, we detailed broad- and fine-scale diversity for the accumulation of physiologically and nutritionally important elements across all five duckweed genera. This variation is associated with dramatic morphological reductions in fundamental plant organs and genome expansions. Thus, disentangling the concurrent effects of dramatic genome size expansions, organ reduction and ecological adaptations will be a great challenge. However, at the more microevolutionary scale, within-species, accession-level variation points to clear promise in mapping alleles responsible for this observed variation.

One might speculate that the observed ionomic changes might be a maladaptive spandrel associated with root loss in derived taxa, but it is hard at this point to identify what the exact trade-off might be; this is for dedicated mechanistic and ecological work on the rootless taxa. Beyond highlighting these enigmatic correlates of root loss and the consequences of organ loss and vestigiality, this work serves to establish phenotypic variation across the ionome at both the fine and broad scale. This serves as a basis for future genomic characterization of causal alleles, in addition to rational development of targeted duckweed lines for both important nutritional and phytoremediation goals.

## SUPPLEMENTARY DATA

Supplementary data are available at *Annals of Botany* online and consist of the following.

Figure S1: raw elemental composition of duckweed whole plants between days 1, 3 and 5 following media change by ICP-MS. Figure S2: outlier accessions with dynamic elemental concentrations over sampling days 1, 3 and 5 after media change. Figure S3: principal component analysis for 11 plant macro- and micronutrients and heavy metals. Figure S4: intensity and direction of correlations between eight elements in 34 duckweed accessions. Table S1: accessions studied in this work, with Landolt codes and locations. Dataset S1: summary elements present in N medium, as measured by ICP-MS. Dataset S2: all ionomics data (in milligrams per kilogram) for 22 elements for 34 accessions on days 1, 3 and 5 post media change quantified by ICP-MS.

mcae012_suppl_Supplementary_Material

mcae012_suppl_Supplementary_Datasets_S1

mcae012_suppl_Supplementary_Datasets_S2

## Data Availability

The data are given as Supplemental Data to the article.

## References

[CIT0001] An D , Li C, Zhou Y, Wu Y, Wang W. 2018. Genomes and transcriptomes of duckweeds. Frontiers in Chemistry 6: 230. 29974050 10.3389/fchem.2018.00230 PMC6019479

[CIT0071] An D , Zhou Y, Li C, et al. 2019. Plant evolution and environmental adaptation unveiled by long-read whole-genome sequencing of *Spirodela*. *Proceedings of the National Academy of Sciences of the United States of America* 116: 18893–18899. 31484765 10.1073/pnas.1910401116 PMC6754600

[CIT0003] Appenroth K , Sree K. 2020. Worldwide genetic resources of duckweed: stock collections. In: Cao X, Fourounjian P, Wang W. eds. The duckweed genomes. Switzerland: Springer, Cham, 39–46.

[CIT0068] Appenroth KJ , Teller S, Horn M. 1996. Photophysiology of turion formation and germination in *Spirodela polyrhiza*. *Biologia Plantarum* 38, 95–106.

[CIT0004] Appenroth KJ , Borisjuk N, Lam E. 2013. Telling duckweed apart: genotyping technologies for the Lemnaceae. Chinese Journal of Applied and Environmental Biology 19: 1–10.

[CIT0005] Appenroth KJ , Sree KS, Böhm V, et al. 2017. Nutritional value of duckweeds (Lemnaceae) as human food. Food Chemistry 217: 266–273. 27664634 10.1016/j.foodchem.2016.08.116

[CIT0006] Appenroth KJ , Sowjanya Sree K, Bog M, et al. 2018. Nutritional value of the duckweed species of the genus *Wolffia* (Lemnaceae) as human food. Frontiers in Chemistry 6: 483. 30420949 10.3389/fchem.2018.00483 PMC6215809

[CIT0007] Arnold BJ , Lahner B, DaCosta JM, et al. 2016. Borrowed alleles and convergence in serpentine adaptation. Proceedings of the National Academy of Sciences of the United States of America 113: 8320–8325. 27357660 10.1073/pnas.1600405113 PMC4961121

[CIT0008] Bog M , Lautenschlager U, Landrock MF, et al. 2015. Genetic characterization and barcoding of taxa in the genera *Landoltia* and *Spirodela* (Lemnaceae) by three plastidic markers and amplified fragment length polymorphism (AFLP). Hydrobiologia 749: 169–182.

[CIT0009] Bog M , Sree KS, Fuchs J, et al. 2020. A taxonomic revision of *Lemna* sect. *Uninerves* (Lemnaceae). Taxon 69: 56–66.

[CIT0010] Boonyapookana B , Upatham ES, Kruatrachue M, Pokethitiyook P, Singhakaew S. 2002. Phytoaccumulation and phytotoxicity of cadmium and chromium in duckweed *Wolffia globosa*. International Journal of Phytoremediation 4: 87–100. 12655803 10.1080/15226510208500075

[CIT0011] Braglia L , Lauria M, Appenroth KJ, et al. 2021. Duckweed species genotyping and interspecific hybrid discovery by tubulin-based polymorphism fingerprinting. Frontiers in Plant Science 12: 625670. 33763089 10.3389/fpls.2021.625670 PMC7982733

[CIT0012] Ceschin S , Abati S, Ellwood NTW, Zuccarello V. 2018. Riding invasion waves: spatial and temporal patterns of the invasive *Lemna minuta* from its arrival to its spread across Europe. Aquatic Botany 150: 1–8.

[CIT0013] Ceschin S , Crescenzi M, Iannelli MA. 2020. Phytoremediation potential of the duckweeds *Lemna minuta* and *Lemna minor* to remove nutrients from treated waters. Environmental Science and Pollution Research 27: 15806–15814. 32088823 10.1007/s11356-020-08045-3

[CIT0014] Crawford DJ , Landolt E, Les DH. 1996. An allozyme study of two sibling species of *Lemna* (Lemnaceae) with comments on their morphology, ecology and distribution. Bulletin of the Torrey Botanical Club 123: 1–6.

[CIT0015] Cross JW. 2017. Duckweed roots: their role in vegetative dispersal. Duckweed Forum 5: 58–59.

[CIT0069] Danku JM , Lahner B, Yakubova E, Salt DE. 2013. Large-scale plant ionomics. *Methods in Molecular Biology* 953:255–276. 23073889 10.1007/978-1-62703-152-3_17

[CIT0016] Ekperusi AO , Sikoki FD, Nwachukwu EO. 2019. Application of common duckweed (*Lemna minor*) in phytoremediation of chemicals in the environment: state and future perspective. Chemosphere 223: 285–309. 30784736 10.1016/j.chemosphere.2019.02.025

[CIT0018] Franceschi VR. 1989. Calcium oxalate formation is a rapid and reversible process in *Lemna minor* L. Protoplasma 148: 130–137.

[CIT0019] Hoang PTN , Schubert V, Meister A, Fuchs J, Schubert I. 2019. Variation in genome size, cell and nucleus volume, chromosome number and rDNA loci among duckweeds. Scientific Reports 9: 3234. 30824726 10.1038/s41598-019-39332-w PMC6397220

[CIT0020] Kadono Y , Iida S. 2022. Identification of a small, spring water-associated duckweed with special reference to the taxonomy of sect. *Uninerves* of the genus *Lemna* (Lemnaceae) in Japan. Acta Phytotaxonomica et Geobotanica 73: 57–65.

[CIT0021] Kara Y , Kara I. 2005. Removal of cadmium from water using duckweed (*Lemna trisulca* L.). International Journal of Agriculture and Biology 7: 660–662.

[CIT0022] Khellaf N , Zerdaoui M. 2009. Growth response of the duckweed *Lemna minor* to heavy metal pollution. Iranian Journal of Environmental Health Science and Engineering 6: 161–166.

[CIT0023] Kimball RT , Crawford DJ, Les DH, Landolt E. 2003. Out of Africa: molecular phylogenetics and biogeography of *Wolffiella* (Lemnaceae). Biological Journal of the Linnean Society 79: 565–576.

[CIT0024] Kirjakov IK , Velichkova KN. 2016. Invasive species *Lemna* L. (Lemnaceae) in the flora of Bulgaria. Periodicum Biologorum 118: 131–138.

[CIT0025] Kurihara D , Mizuta Y, Sato Y, Higashiyama T. 2015. ClearSee: a rapid optical clearing reagent for whole-plant fluorescence imaging. Development (Cambridge, England) 142: 4168–4179. 26493404 10.1242/dev.127613 PMC4712841

[CIT0026] Lahive E , O’Callaghan MJA, Jansen MAK, O’Halloran J. 2011. Uptake and partitioning of zinc in Lemnaceae. Ecotoxicology 20: 1992–2002. 21755349 10.1007/s10646-011-0741-y

[CIT0027] Lam E , Michael TP. 2022. *Wolffia*, a minimalist plant and synthetic biology chassis. Trends in Plant Science 27: 430–439. 34920947 10.1016/j.tplants.2021.11.014

[CIT0028] Landolt E. 1980. Key to the determination of taxa within the family of Lemnaceae. Veröffentlichungen des Geobotanischen Institutes der Eidgenössisch.Technische Hochschule, Stiftung Rübel, Zürich 70: 13–21.

[CIT0029] Landolt E. 1986. Biosystematic investigations in the family of duckweeds (Lemnaceae). Vols 1 and 2. Zürich: Geobotanisches Institut der ETH.

[CIT0030] Landolt E. 1998. *Lemna yungensis*, a new duckweed species from rocks of the Andean Yungas in Bolivia Andean Yungas in Bolivia. Bulletin of the Geobotanical Institute ETH 64: 15–21.

[CIT0031] Landolt E , Kandeler R. 1987. The family of Lemnaceae: a monographic study, Vol. 2. Zurich: Geobotanischen Institutes der ETH.

[CIT0032] Leblebici Z , Aksoy A, Duman F. 2010. Influence of nutrient addition on growth and accumulation of cadmium and copper in *Lemna gibba*. Chemical Speciation and Bioavailability 22: 157–164.

[CIT0033] Les DH , Crawford DJ. 1999. *Landoltia* (Lemnaceae), a new genus of duckweeds. Novon 9: 530–533.

[CIT0034] Les DH , Crawford DJ, Landolt E, Gabel JD, Kimball RT. 2002. Phylogeny and systematics of Lemnaceae, the duckweed family. Systematic Botany 27: 221–240.

[CIT0035] Liu Y , Sanguanphun T, Yuan W, Cheng JJ, Meetam M. 2017. The biological responses and metal phytoaccumulation of duckweed *Spirodela polyrhiza* to manganese and chromium. Environmental Science and Pollution Research International 24: 19104–19113. 28660513 10.1007/s11356-017-9519-y

[CIT0036] Ma X , Zeng J, He Y, et al. 2023. Cadmium accumulation in duckweed relates to pH and oxalate synthesis in Cd shock. Journal of Aquatic Plant Management 61: 55–62.

[CIT0037] Mazen AMA , Zhang D, Franceschi VR. 2003. Calcium oxalate formation in *Lemna minor*: physiological and ultrastructural aspects of high capacity calcium sequestration. New Phytologist 161: 435–448. 10.1111/j.1469-8137.2004.00923.x 33873511

[CIT0038] McKenna S , Meyer M, Gregg C, Gerber S. 2016. s-CorrPlot: an interactive scatterplot for exploring correlation. Journal of Computational and Graphical Statistics 25: 445–463.

[CIT0039] Michael TP , Ernst E, Hartwick N, et al. 2020. Genome and time-of-day transcriptome of Wolffia *a*ustraliana link morphological extreme minimization with un-gated plant growth. Genome Research 31: 225–238. 33361111 10.1101/gr.266429.120 PMC7849404

[CIT0040] Mkandawire M , Dudel E. 2007. Are *Lemna* spp. effective phytoremediation agents? Bioremediation, Biodiversity and Bioavailability 1: 56–71.

[CIT0041] Naumann B , Eberius M, Appenroth KJ. 2007. Growth rate based dose–response relationships and EC-values of ten heavy metals using the duckweed growth inhibition test (ISO 20079) with *Lemna minor* L. clone St. Journal of Plant Physiology 164: 1656–1664. 17296247 10.1016/j.jplph.2006.10.011

[CIT0042] Njambuya J , Stiers I, Triest L. 2011. Competition between *Lemna minuta* and *Lemna minor* at different nutrient concentrations. Aquatic Botany 94: 158–164.

[CIT0043] Paolacci S , Harrison S, Jansen MAK. 2016. A comparative study of the nutrient responses of the invasive duckweed *Lemna minuta*, and the native, co-generic species *Lemna minor*. Aquatic Botany 134: 47–53.

[CIT0044] Park H , Park JH, Lee Y, et al. 2021. Genome of the world’s smallest flowering plant, *Wolffia australiana*, helps explain its specialized physiology and unique morphology. Communications Biology 4: 900. 34294872 10.1038/s42003-021-02422-5 PMC8298427

[CIT0045] Prasad MNV , Malec P, Waloszek A, Bojko M, Strzalka K. 2001. Physiological responses of *Lemna trisulca* L. (duckweed) to cadmium and copper bioaccumulation. Plant Science 161: 881–889.

[CIT0046] Ren Q , Xu Z, Xue Y, et al. 2022. Mechanism of calcium signal response to cadmium stress in duckweed. Plant Signaling and Behavior 17: 2119340. 36102362 10.1080/15592324.2022.2119340 PMC9481097

[CIT0070] R Development Core Team. 2023. *R: a Language and Environment for Statistical Computing*. R Foundation for Statistical Computing, Vienna, Austria. https://www.R-project.org/.

[CIT0047] RStudio Team. 2020. RStudio: integrated development for R. Boston, MA: R Studio, Inc.

[CIT0048] Schreinemakers WAC. 1986. The interaction Cd-absorption and Cd-compartmentation in *Wolffiella gladiata*. Acta Botanica Neerlandica 35: 23–34.

[CIT0049] Tippery NP , Les DH, Crawford DJ. 2015. Evaluation of phylogenetic relationships in Lemnaceae using nuclear ribosomal data. Plant Biology (Stuttgart, Germany) 17: 50–58. 24942778 10.1111/plb.12203

[CIT0050] Van Dam RA , Hogan AC, McCullough CD, Houston MA, Humphrey CL, Harford AJ. 2010. Aquatic toxicity of magnesium sulfate, and the influence of calcium, in very low ionic concentration water. Environmental Toxicology and Chemistry 29: 410–421. 20821461 10.1002/etc.56

[CIT0051] Van Steveninck RFM , Van Steveninck ME, Fernando DR. 1992. Heavy-metal (Zn, Cd) tolerance in selected clones of duck weed (*Lemna minor*). Plant and Soil 146: 271–280.

[CIT0052] Verma R , Suthar S. 2015. Lead and cadmium removal from water using duckweed – *Lemna gibba* L.: impact of pH and initial metal load. Alexandria Engineering Journal 54: 1297–1304.

[CIT0053] Volkova PA , Nachatoi VA, Bobrov AA. 2023. Hybrid between *Lemna minor* and *L. turionifera* (*L.* × *japonica*, Lemnaceae) in East Europe is more frequent than parental species and poorly distinguishable from them. Aquatic Botany 184: 103593.

[CIT0054] Walsh E , Paolacci S, Burnell G, Jansen MAK. 2020. The importance of the calcium-to-magnesium ratio for phytoremediation of dairy industry wastewater using the aquatic plant *Lemna minor* L. International Journal of Phytoremediation 22: 694–702. 31910655 10.1080/15226514.2019.1707478

[CIT0055] Wang W. 1991. Literature review on higher plants for toxicity testing. Water, Air, and Soil Pollution 59: 381–400.

[CIT0056] Wang W , Wu Y, Yan Y, Ermakova M, Kerstetter R, Messing J. 2010. DNA barcoding of the Lemnaceae, a family of aquatic monocots. BMC Plant Biology 10: 205. 20846439 10.1186/1471-2229-10-205 PMC2956554

[CIT0057] Wang W , Kerstetter RA, Michael TP. 2011. Evolution of genome size in duckweeds (Lemnaceae). Journal of Botany 2011: 570319.

[CIT0058] Ware A , Jones DH, Flis P, et al. 2023. Loss of ancestral function in duckweed roots is accompanied by progressive anatomical simplification and a re-distribution of nutrient transporters. Current Biology 33: 1795–1802.e4. 36990089 10.1016/j.cub.2023.03.025

[CIT0059] Wong ELY , Hiscock SJ, Filatov DA. 2022. The role of interspecific hybridisation in adaptation and speciation: insights from studies in *Senecio*. Frontiers in Plant Science 13: 907363–907373. 35812981 10.3389/fpls.2022.907363 PMC9260247

[CIT0060] Xie WY , Huang Q, Li G, Rensing C, Zhu YG. 2013. Cadmium accumulation in the rootless macrophyte *Wolffia globosa* and its potential for phytoremediation. International Journal of Phytoremediation 15: 385–397. 23488004 10.1080/15226514.2012.702809

[CIT0061] Xu S , Stapley J, Gablenz S, et al. 2019. Low genetic variation is associated with low mutation rate in the giant duckweed. Nature Communications 10: 1243. 10.1038/s41467-019-09235-5 PMC6423293 30886148

[CIT0062] Yang GL , Fang Y, Xu YL, et al. 2018a. Frond transformation system mediated by *Agrobacterium tumefaciens* for *Lemna minor*. Plant Molecular Biology 98: 319–331. 30298427 10.1007/s11103-018-0778-x

[CIT0063] Yang J , Li G, Hu S, et al. 2018b. A protocol for efficient callus induction and stable transformation of *Spirodela polyrhiza* (L.) Schleiden using *Agrobacterium tumefaciens*. Aquatic Botany 151: 80–86.

[CIT0064] Yang J , Zhao X, Li G, Hu S, Hou H. 2021. Frond architecture of the rootless duckweed *Wolffia globosa*. BMC Plant Biology 21: 1–10. 34416853 10.1186/s12870-021-03165-5 PMC8377843

[CIT0065] Zayed A , Gowthaman S, Terry N. 1998. Phytoaccumulation of trace elements by wetland plants: I. Duckweed. Journal of Environmental Quality 27: 715–721.

[CIT0066] Zhang X , Zhao FJ, Huang Q, Williams PN, Sun GX, Zhu YG. 2009. Arsenic uptake and speciation in the rootless duckweed *Wolffia globosa*. The New Phytologist 182: 421–428. 19210724 10.1111/j.1469-8137.2008.02758.x

[CIT0067] Zheng MM , Feng D, Liu HJ, Yang GL. 2023. Subcellular distribution, chemical forms of cadmium and rhizosphere microbial community in the process of cadmium hyperaccumulation in duckweed. The Science of the Total Environment 859: 160389. 36423841 10.1016/j.scitotenv.2022.160389

